# Volumetric measurement of paranasal sinuses and its clinical significance in pituitary neuroendocrine tumors operated using an endoscopic endonasal approach

**DOI:** 10.3389/fneur.2023.1162733

**Published:** 2023-03-30

**Authors:** Masato Nakaya, Ryota Tamura, Kento Takahara, Takumi Senuma, Keisuke Yoshida, Yohei Kitamura, Ryo Ueda, Masahiro Toda

**Affiliations:** ^1^Department of Neurosurgery, Keio University School of Medicine, Tokyo, Japan; ^2^Department of Neurosurgery, Mihara Memorial Hospital, Isesaki-shi, Japan

**Keywords:** pituitary neuroendocrine tumor, endoscopic endonasal surgery, paranasal sinus, volumetric analysis, sphenoid sinus

## Abstract

**Objective:**

Endoscopic endonasal surgery (EES) for deep intracranial lesions has gained popularity following recent developments in endoscopic technology. The operability of invasive pituitary neuroendocrine tumors (PitNETs) depends on the anatomy of the nasal cavity and paranasal sinus. This study aimed to establish a simple volume reconstruction algorithm of the nasal cavity and paranasal sinus. Additionally, this is the first study to demonstrate the relationship between the segmentation method and the clinical significance in patients with PitNET.

**Methods:**

Pre-and postoperative tumor volumes were analyzed in 106 patients with primary (new-onset) PitNETs (80 nonfunctioning and 26 functioning) who underwent EES. The efficiency and accuracy of the semiautomatic segmentation with manual adjustments (SSMA) method was compared with other established segmentation methods for volumetric analysis in the nasal cavity and paranasal sinuses. Correlations between the measured nasal cavity and paranasal sinus volumes and the extent of tumor removal were evaluated.

**Results:**

The SSMA method yielded accurate and time-saving results following the volumetric analyses of nasal cavity and paranasal sinuses with complex structures. Alternatively, the manual and semiautomatic segmentation methods proved time-consuming and inaccurate, respectively. The sphenoid sinus volume measured by SSMA was significantly correlated with the extent of tumor removal in patients with nonfunctioning Knosp grade 3 and 4 PitNET (*r* = 0.318; *p* = 0.015).

**Conclusion:**

The volume of sphenoid sinus potentially could predict the extent of resection due to better visualization of the tumor for PitNETs with CS invasion.

## 1. Introduction

Transnasal-transphenoidal surgery has been performed using a microscope since the 1960s (1, 2) followed by the use of a transnasal endoscope in the 1990s (3). Endoscopic surgery successfully improved the surgical outcomes by enabling the observation of areas that were considered as blind spots during microscopic surgery (4). Currently, endonasal endoscopic surgery (EES) enables surgical exposure from the olfactory groove to the craniovertebral junction in the midline and to the infratemporal region and jugular fossa laterally (5).

However, the difficulty of ESS depends on the gross anatomy of the nasal cavity and paranasal sinuses. Pituitary neuroendocrine tumors (PitNETs) that laterally invade the cavernous sinus (CS) are difficult to access *via* EES, resulting in low removal rates (6–9). Previously, we demonstrated the relationship between pneumatization of the lateral recess in the sphenoid sinus and the extent of tumor resection in CS (10, 11). Furthermore, a two-dimensional method using various intranasal anatomical structures was established to predict the surgical field around the sella turcica (12). Preoperative simulation, taking the nasal and paranasal sinus structures into consideration, can improve the efficiency and optimize the effect of the surgical procedure.

The quantification of complex pneumatization in the nasal cavity and paranasal sinus is challenging; nonetheless, they should be evaluated as a three-dimensional (3D) anatomy model. In recent years, tumor volume evaluations have shifted from linear measurements to volumetric analysis. The present study successfully established a simple volume reconstruction algorithm of the nasal cavity and paranasal sinus. Furthermore, this is the first study to demonstrate the relationship between the segmentation method and the clinical significance in patients with PitNET.

## 2. Materials and methods

### 2.1. Patients

A total of 106 patients with primary (new-onset) PitNETs who underwent surgery *via* EES from October 2012 to September 2022 at Keio University Hospital were retrospectively evaluated. The SYNAPSE VINCENT imaging system (Fujifilm Medical Co., Tokyo, Japan) was used for volumetric analysis. CS invasion was reported according to the Knosp criteria (13), and only those with Knosp grades 3 and 4 were included to evaluate the relationship between the lateral tumor extension and the simulation method used in the study. This study was approved by the Institutional Review Board (reference number: 20130379). Informed consent was obtained from all participants included in the study.

### 2.2. Comparison of the volume measurement methods used in the nasal cavity and paranasal sinus

**Figure 1:**
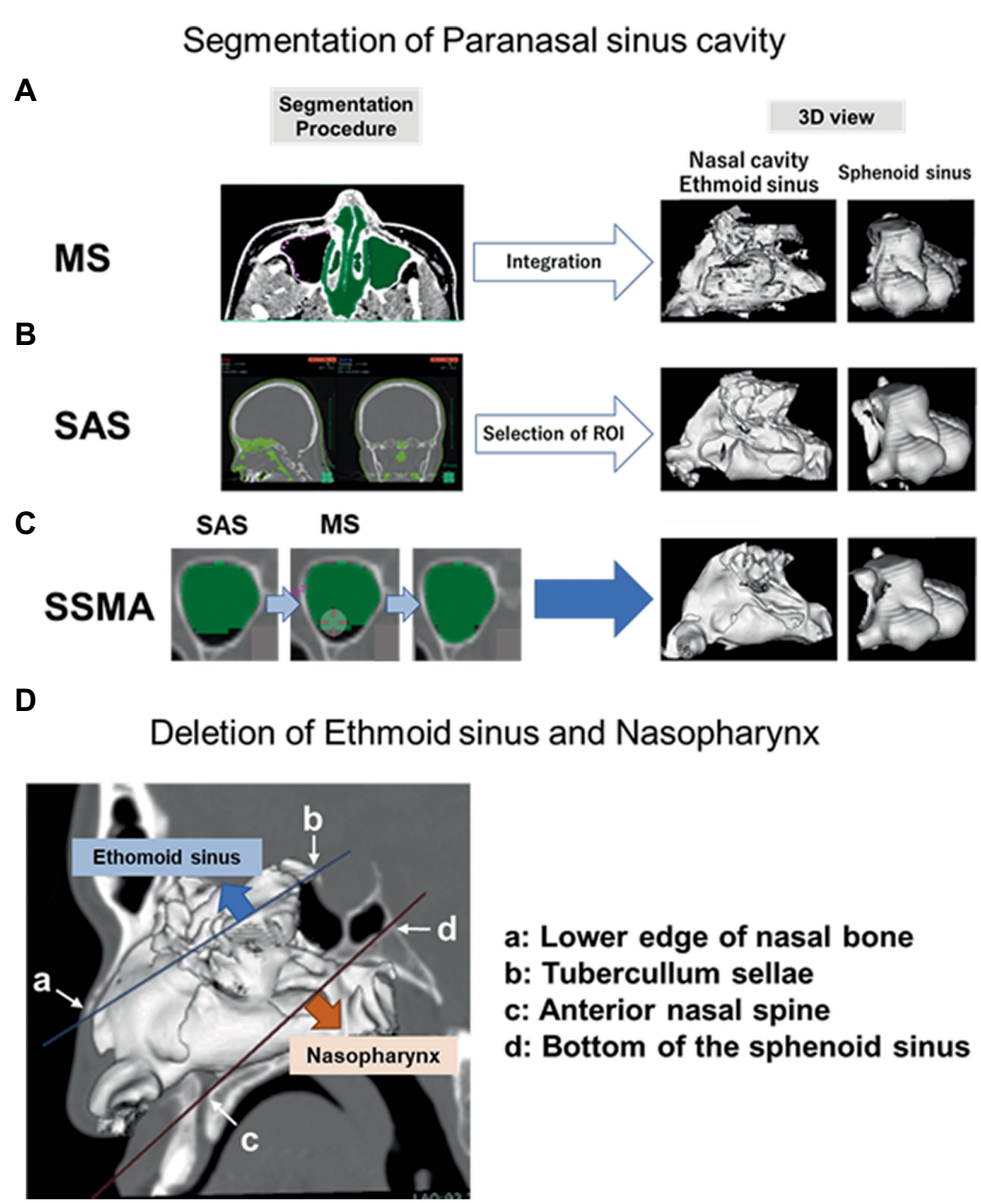
Procedures for each surgical simulation method. Regions determined by MS, SAS, and SSMA. Detailed procedures of each method (left panel) and 3D models of the nasal cavity, ethmoid sinus, and sphenoid sinus (right panel) are shown. **(A)** In the MS procedure, we drew a region of interest around the lesion boundaries on each slice where the lesion was observed. The area was multiplied by the slice thickness to calculate the volume of the lesion within that slice, and the volumes from all slices were added to yield the total lesion volume. **(B)** A set of voxels was extracted from the preoperative CT in the value range of [−1,500−−200] HU (air density) in the SAS. **(C)** Unextracted regions in the nasal cavity and each paranasal sinus were manually collected after performing the same procedures in SSMA. **(D)** Deletion areas of the ethmoid sinus and nasopharynx on the sagittal section are shown. MS, manual segmentation; SAS, semiauto segmentation; and SSMA, semiautomatic segmentation with manual adjustments.

The efficiency and accuracy of three methods (Figure 1) were evaluated as follows.

#### 2.2.1. Manual segmentation

- The boundaries of the nasal cavity and paranasal sinuses were drawn to every 3 mm slice in the axial section of the preoperative computed tomography (CT; Figure 1A).
- The volume was calculated by integrating them as a 3D object.

#### 2.2.2. Semiautomatic segmentation

- From the preoperative CT, a set of voxels was extracted in the CT value range of (−1,500 ~ −200) HU, which is the air density (Figure 1B).
- The volumes of the nasal cavity and all the paranasal sinuses were calculated by manually deleting the ineligible anatomical structures. The nasal cavity and each paranasal sinus (maxillary, sphenoidal, and ethmoidal sinus) were selected. The nasal cavity was defined as the area bounded by two lines in the sagittal section. One line connected the lower edge of the nasal bone to the tuberculum sellae, and the other connected the anterior nasal spine to the bottom of the sphenoid sinus; the area below this line was defined as the nasopharyngeal region. The frontal sinus was deleted from the segmentation because it did not contribute to the surgical maneuver for PitNET.

#### 2.2.3. Semiautomatic segmentation with manual adjustments

Unextracted regions in the nasal cavity and each paranasal sinus were manually collected after using the same procedures as in the semiautomatic segmentation (SAS) method (B; Figure 1C).

### 2.3. Measurements and data analysis

Correlations between the nasal cavity and paranasal sinus volumes and the physical characteristics (height, weight, and body surface area) of the patients were evaluated to elucidate the differences among individuals. Next, correlations between the nasal cavity and paranasal sinus volumes and the extent of tumor resection were investigated. Gadolinium-enhanced T1-weighted images were used to evaluate the pre-and post-operative tumor volumes. Semiautomatic segmentation with manual adjustment (SSMA) was adopted to measure the volumes of the nasal cavities and paranasal sinuses.

### 2.4. Statistical analysis

Receiver operating characteristic (ROC) curves were constructed to determine the optimal cut-off values in order to predict the extent of tumor resection. Subsequently, each variable was analyzed as a dichotomous variable according to the optimal cut-off value. The Mann–Whitney U test was used to compare the extent of tumor resection. Pearson’s correlation analysis was performed to analyze the correlation between two variables. The coefficient of variation (CV), which is the standard deviation divided by the arithmetic mean, was used to compare the variabilities in populations with different means. All statistical analyses were conducted using SPSS statistics 28.0.0.0 (IBM, NY, United States) and Prism 8 (Graphpad software corporation, CA, United States). *p* < 0.05 was considered statistically significant.

## 3. Results

### 3.1. Patient characteristics

The 106 patients with PitNET were classified into five groups based on the Knosp tumor grade (Grade 0, *n* = 5; Grade 1, *n* = 10; Grade 2, *n* = 17; Grade 3, *n* = 38; and Grade 4, *n* = 36), as shown in Table 1. The mean age of the patients (52 men and 54 women) was 56.1 years (range, 17–85 years). The mean pre-and post-operative tumor volumes were 10.26 mL (range, 0.050–71.09) and 0.91 mL (range, 0–11.94), respectively. Based on the blood test results, 80 patients had a nonfunctioning pituitary, and 26 had functioning pituitary glands.

**Table 1:** Distribution of patients based on the tumor grade.

Knosp Grade^#1^	Nonfunctioning	Functioning	Total
0	2	3	5
1	5	5	10
2	14	3	17
3	30	8	38
4	29	7	36
Total	80	26	106

^#1^Cavernous sinus (CS) invasion was evaluated using the new Knosp classification. The Knosp grading system includes five categories: (1) Grade 0, no invasion with all of the lesion medial to the cavernous carotid artery; (2) Grade 1, invasion extending to, but not past, the medial aspect of the cavernous carotid artery; (3) Grade 2, invasion extending to, but not past, the lateral aspect of the cavernous carotid artery; (4) Grade 3, invasion past the lateral aspect of the cavernous carotid artery, but not completely filling the CS; and (5) Grade 4, tumor completely filling the CS both medial and lateral to the cavernous carotid artery.

### 3.2. Establishment of volume measurement method

**Figure 2:**
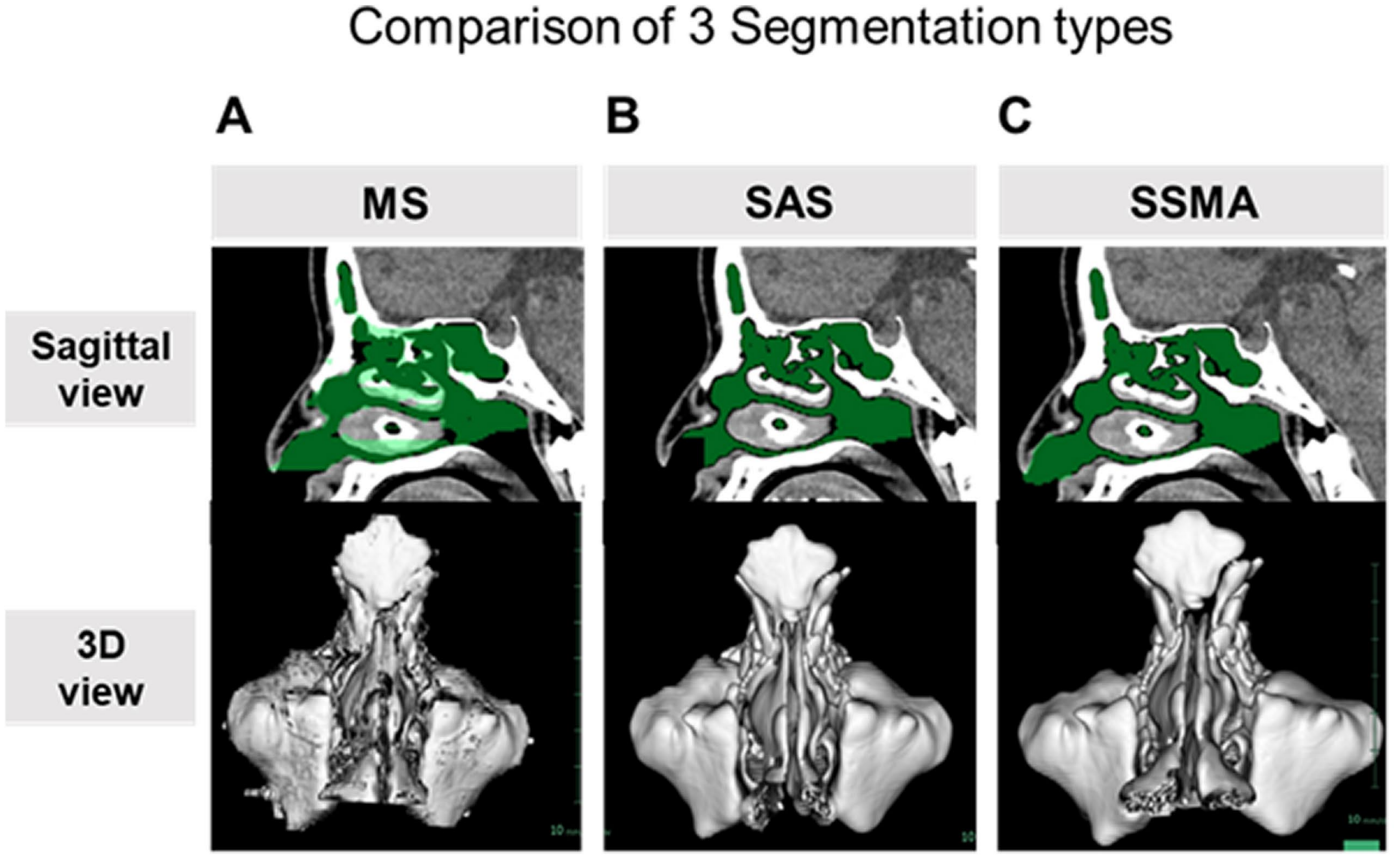
Comparison of the three surgical simulation methods. The extracted areas (green) in the nasal cavity and paranasal sinuses in the sagittal section (upper panel) and 3D models (lower panel) using the MS **(A)**, SAS **(B)**, and SSMA **(C)** methods. MS, manual segmentation; SAS, semiauto segmentation; and SSMA, semiautomatic segmentation with manual adjustments.

The desired structures could be manually segmented for the complex shapes and uncertain borders between different tissues. Although the surfaces of the maxillary sinus and sphenoidal sinus were reasonably clear, most of the surface images in manual segmentation (MS) were not fine compared to those from the other two methods (Figure 2A). Furthermore, the MS method was found to be extremely time-consuming and cumbersome. Thus, SAS could be a time-saving alternative to MS in cases where fully automatic segmentation algorithms failed to perform at the desired levels of accuracy. A significant difference was observed between the semiautomatic and manual modalities in terms of the mean segmentation time (MS, 46.3 min/case; SAS, 13.6 min/case, *p* < 0.001). However, the output boundaries between air and the thin bone were not entirely accurate after using the SAS method (Figure 2B; Table 2).

**Table 2:** Measurement time for each method.

Segmentation method	Time (minute, mean ± SD) (min-max)
Manual segmentation	46.3 ± 8.9 (41–58)
Semiautomatic segmentation	13.6 ± 1.9 (10–16.5)
SSMA	29.2 ± 9.2 (21–50)

Mean time required by each method to complete a procedure. SSMA, semiautomatic segmentation with manual adjustments.

Likewise, a significant difference between SSMA and SAS (*p* < 0.001) was observed in terms of the segmentation time, but it was significantly reduced (*p* = 0.002) compared to MS (SSMA, 29.2 min/case; Table 2). Furthermore, each paranasal sinus could be separately analyzed *via* the SSMA method compared to the SAS method (Figure 2C).

### 3.3. Clinical significance of the SSMA method

There were individual differences in maximum width (CV = 0.248) compared with maximum depth (CV = 0.103) of the sphenoid sinus. The maximum width was significantly correlated with the sphenoid sinus volume (*p* < 0.001; *r* = 0.818). The sphenoid sinus volume also differed among each patient when compared to the other sinuses (CV = 0.605). The volume of the sphenoid sinus was more strongly correlated with the height (*p* < 0.001) than with the body surface area (*p* = 0.008) of the patient (Table 3). No significant differences were observed between other variables and the anatomical structures.

**Table 3:** Pearson’s correlation coefficient for the general characteristics of the patients.

Anatomical structure	Height	Weight	Body surface area
Nasal cavity and paranasal sinuses	***r* = 0.477**	*r* = 0.192	***r* = 0.326**
	***p* < 0.001**	*p* = 0.098	***p* = 0.004**
Sphenoid sinus	***r* = 0.455**	*r* = 0.176	***r* = 0.306**
	***p* < 0.001**	*p* = 0.131	***p* = 0.008**

Correlation coefficients between the volume of each anatomical structure and patient’s general characteristics (height, weight, and body surface area) are shown. The bold values means values with significant difference.

**Figure 3:**
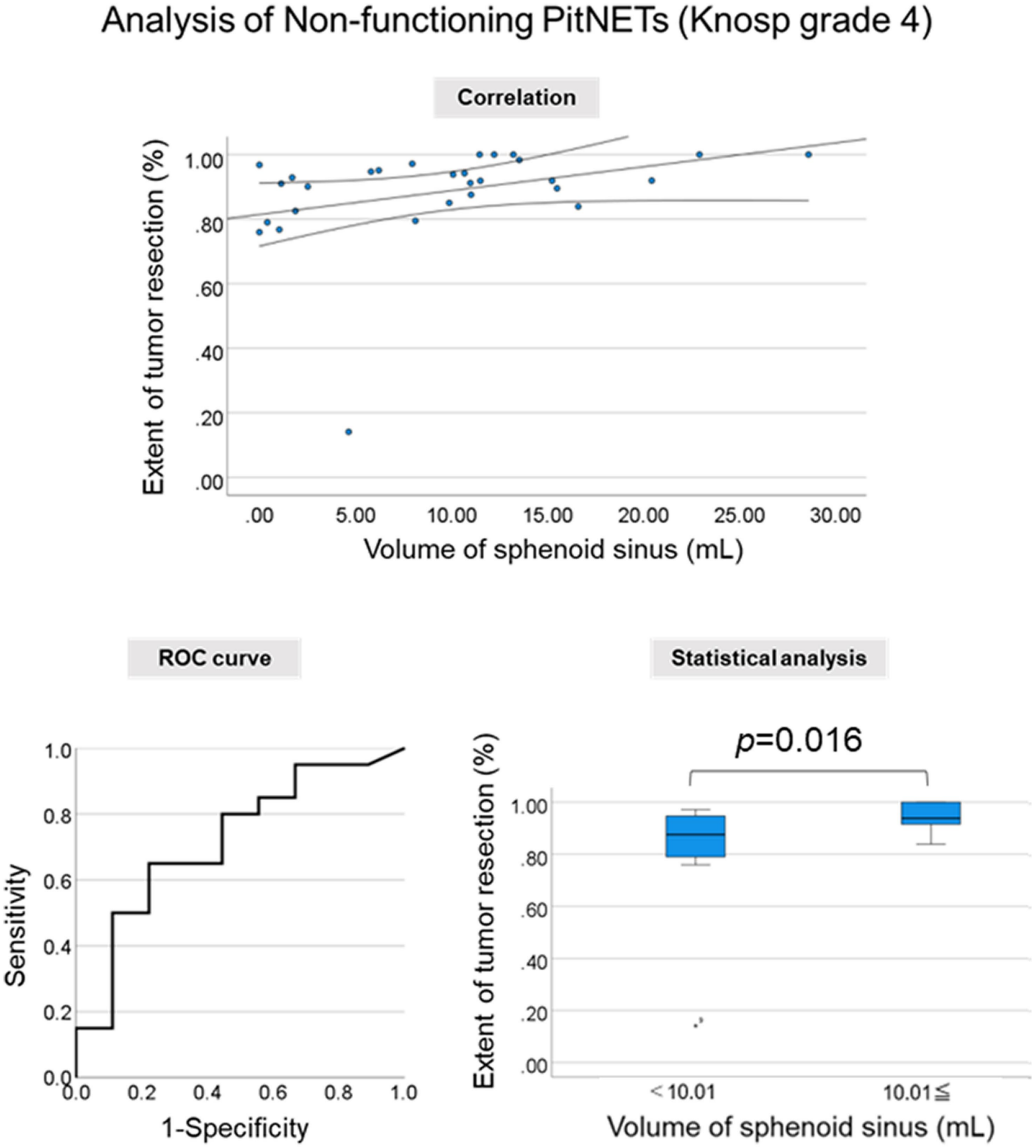
Clinical significance of the segmentation method. Correlation between the sphenoid sinus volume and the extent of tumor resection. Correlation plot **(Upper Middle Panel)**, receiver operating characteristic (ROC) curve **(Lower Left Panel)**, and extent of tumor resection in two groups divided by the cut-off value in nonfunctioning Knosp grade 4 PitNETs **(Lower Right Panel)**.

In patients with grade 3 and 4 PitNET according to Knosp scale, a significant correlation was observed between the sphenoid sinus volume and the extent of tumor resection (*p* = 0.033; *r* = 0.248). Similarly, a significant correlation was seen in the nonfunctioning grade 3 and 4 PitNET subgroup (*p* = 0.015; *r* = 0.318); this tendency was stronger in the nonfunctioning grade 4 PitNET subgroup (*p* = 0.008; *r* = 0.488; Figure 3; Table 4).

**Table 4:** Pearson’s correlation coefficient for the extent of tumor resection.

	Anatomical structure	Extent of tumor resection
Grade 3 and 4	Nasal cavity and paranasal sinuses	*r* = 0.172
		*p* = 0.198
	Sphenoid sinus	***r* = 0.318**
		***p* = 0.015**
Grade 3	Nasal cavity and paranasal sinuses	*r* = −0.121
		*p* = 0.524
	Sphenoid sinus	*r* = 0.039
		*p* = 0.837
Grade 4	Nasal cavity and paranasal sinuses	***r* = 0.411**
		***p* = 0.030**
	Sphenoid sinus	***r* = 0.488**
		***p* = 0.008**

Correlation coefficients between the volume of each measurement area and extent of tumor resection in nonfunctioning Knosp Grade 3and 4 PitNETs. Subgroup analysis of nonfunctioning Knosp Grade 3 and 4 PitNETs is also shown. The bold values means values with significant difference.

Importantly, the sphenoid sinus volume was more strongly associated with the extent of tumor resection in the nonfunctioning grade 4 PitNET compared with the maximum width of the sphenoid sinus (*p* = 0.029, *r* = 0.405). This result suggested that the volumetric analysis of sphenoid sinus with complex structures may be more important for the surgical procedures of EES compared with linear measurement.

To evaluate the association with the extent of tumor resection, a cut-off value greater than 10.01 mL for the sphenoid sinus volume was used to include the highest true-positive rates and lowest false-positive rates. A significant difference in the extent of tumor resection in patients with nonfunctioning Knosp grade 4 PitNET was observed when the cut-off values were used (*p* = 0.016).

## 4. Discussion

Volumetric analysis has been performed using the Cavalieri principle based on the airflow dynamics (14–18), and MS was generally reported to be highly accurate (19–21). However, MS is a very time-consuming method. Although the fully automatic and SAS method appear to be more effective, considering the time-saving potential, it requires complex computational resources; moreover, it is not easy to obtain satisfactory results with this method (22, 23).

Various segmentation methods have been used to perform the volumetric analysis of the head and nasal cavities. Breakey et al. investigated the optimal segmentation method by comparing the results of fully automatic, semiautomatic, and MS methods for intracranial volume analysis (24). The fully automatic method is not easy to use because it takes considerable time to master the technique and obtain the results with the same precision as other methods. Although the volume of the nasal cavity could be previously analyzed using SAS, the method lacked the precision to analyze the complex structures (25, 26). The present study demonstrated that the results of SAS were not accurate for complex structures and for the boundary between air and the thin bone in the nasal cavity. Lentzen et al. reported that the sphenoid sinus volume could be measured accurately and conveniently using SAS with MS correction, which was similar to the SSMA method proposed in this study (27, 28). However, the procedure used for SSMA was simpler compared to the other reported methods; SSMA was found to be highly practical, efficient, and accurate in the present study.

To the best of our knowledge, this is the first study to demonstrate the relationship between the segmentation method and the clinical significance in patients with PitNET. In the present study, the volume of the sphenoid sinus was strongly associated with the extent of tumor resection in nonfunctioning PitNETs with CS invasion. This result suggests that a 3D volumetric analysis of the gross anatomical variation is vital for surgical simulation. The paranasal sinus volume analyzed by SSMA might prove useful for surgeons to estimate the difficulty of surgery. The cut-off value may be also important for preoperative simulation. For the Knosp grade 4 PitNET with narrow sphenoid sinus (< cut-off value), we need to prepare special surgical instruments, such as angled endoscopes and curved suction tubes. Furthermore, this study demonstrated that the sphenoid sinus volume was strongly associated with the height of the patient. This information will aid in predicting the size of the sphenoid sinus. However, no significant correlations were observed in patients with functioning PitNETs, which might be attributed to the small number of cases. Furthermore, it is known that functioning PitNETs are difficult to remove due to the different nature of the tumor (29, 30).

The main limitation of this study was the small number of cases assessed. Future studies analyzing a large number of patients are warranted to confirm these findings. There are many other factors involved in the removal of a tumor, including the nature of the tumor itself, as described above. Tumor characteristics, including fibrous tissue, tumor vascularity, and extent of invasion, must be considered to evaluate the difficulty of the EES procedures. Surgical instruments are also associated with the extent of tumor resection. For instance, angled endoscopes allow for more lateral and superior vision. Actually, in this study, a significant correlation was seen between the sphenoid sinus volume and the extent of resection in the nonfunctioning grade 3 and 4 PitNET; this tendency was stronger in the grade 4 PitNETs subgroup. Lateral tumor component in the Knosp grade 3 PitNET can be often removed using angled endscopes and curved suction tubes. However, lateral tumor component in the Knosp grade 4 PitNET is more difficult to be removed using any surgical instruments. Preoperative surgical simulation considering various types of instruments is important for the practical clinic. Wide opening of the anterior wall of the sphenoid sinus was performed as the routine surgical procedure in our hospital. However, detailed size of opened anterior wall was not measured in this study, which is a crucial point to be discussed. We will conduct this analysis as the future research. Finally, a prospective study using this simulation method is needed to generalize the results of the current study.

## 5. Conclusion

The present study successfully established a simple volume reconstruction algorithm of the nasal cavity and paranasal sinus. The volume of sphenoid sinus potentially could predict the extent of resection due to better visualization of the tumor for PitNETs with CS invasion.

Permission must be obtained for use of copyrighted material from other sources (including the web). Please note that it is compulsory to follow figure instructions.

## Data availability statement

The original contributions presented in the study are included in the article/supplementary material, further inquiries can be directed to the corresponding author.

## Ethics statement

The studies involving human participants were reviewed and approved by Keio University Ethics Committee. Written informed consent to participate in this study was provided by the participants’ legal guardian/next of kin.

## Author contributions

MN and RT: conception and design and manuscript writing. MN, KT, KY, and RT: collection and/or assembly of data. MN, KT, and RT: data analysis and interpretation. YK, RU, and MT: manuscript reviewing. MT: supervision. All authors contributed to the article and approved the submitted version.

## Conflict of interest

The authors declare that the research was conducted in the absence of any commercial or financial relationships that could be construed as a potential conflict of interest.

## Publisher’s note

All claims expressed in this article are solely those of the authors and do not necessarily represent those of their affiliated organizations, or those of the publisher, the editors and the reviewers. Any product that may be evaluated in this article, or claim that may be made by its manufacturer, is not guaranteed or endorsed by the publisher.
